# Impact of Obesity-Induced Inflammation on Cardiovascular Diseases (CVD)

**DOI:** 10.3390/ijms22094798

**Published:** 2021-04-30

**Authors:** Gopi Battineni, Getu Gamo Sagaro, Nalini Chintalapudi, Francesco Amenta, Daniele Tomassoni, Seyed Khosrow Tayebati

**Affiliations:** 1 Telemedicine and Telepharmacy Centre, School of Medicinal and Health Products Sciences, University of Camerino, 62032 Camerino, Italy; gopi.battineni@unicam.it (G.B.); getugamo.sagaro@unicam.it (G.G.S.); nalini.chintalapudi@unicam.it (N.C.); francesco.amenta@unicam.it (F.A.); 2 School of Medicinal Sciences and Health Products, University of Camerino, 62032 Camerino, Italy; 3 School of Biosciences and Veterinary Medicine, University of Camerino, 62032 Camerino, Italy; daniele.tomassoni@unicam.it

**Keywords:** obesity, inflammation, cardiovascular diseases, inflammatory cytokines, metabolic syndrome

## Abstract

Overweight and obesity are key risk factors of cardiovascular disease (CVD). Obesity is currently presented as a pro-inflammatory state with an expansion in the outflow of inflammatory cytokines, such as interleukin-6 (IL-6) and tumor necrosis factor-alpha (TNF-α), alongside the expanded emission of leptin. The present review aimed to evaluate the relationship between obesity and inflammation and their impacts on the development of cardiovascular disease. A literature search was conducted by employing three academic databases, namely PubMed (Medline), Scopus (EMBASE), and the Cumulative Index to Nursing and Allied Health Literature (CINAHL). The search presented 786 items, and by inclusion and exclusion filterers, 59 works were considered for final review. The Newcastle–Ottawa Scale (NOS) method was adopted to conduct quality assessment; 19 papers were further selected based on the quality score. Obesity-related inflammation leads to a low-grade inflammatory state in organisms by upregulating pro-inflammatory markers and downregulating anti-inflammatory cytokines, thereby contributing to cardiovascular disease pathogenesis. Because of inflammatory and infectious symptoms, adipocytes appear to instigate articulation and discharge a few intense stage reactants and carriers of inflammation. Obesity and inflammatory markers are strongly associated, and are important factors in the development of CVD. Hence, weight management can help prevent cardiovascular risks and poor outcomes by inhibiting inflammatory mechanisms.

## 1. Introduction

Obesity is associated, in part, with poor dietary and lifestyle decisions. Inactive lifestyles are becoming more prominent worldwide, leading to clinical, social, and monetary challenges [1]. Individuals who suffer from obesity can understand how difficult it is to lose weight through physical activity and diet only. A key region of the brain, the hypothalamus, regulates body weight and feeding behaviors [2]. Moreover, obesity is associated with hypothalamic injury in rodents [3]. The idea behind this is that, an eating regiment high in fat, which causes weight gain, can prompt hypothalamus damage. Among the potential mechanisms of these injuries, irritation is a significant one, but other mechanisms are conceivable as well.

Chronic inflammation related to obesity is linked to an increased risk of various medical problems, including cancer and dementia [4,5,6]. This issue is likely connected to different incendiary markers and the hormone leptin, which is secreted by fat cells [7,8,9].

Obesity increases the risk of cardiovascular sickness and certain diseases; moreover, abdominal obesity is one of the conditions for metabolic syndrome (MetS) [10]. MetS is a group of components that occur together to increase one’s risk of cardiovascular sickness; other underlying components include insulin resistance, hyperglycemia with prediabetes or type 2 diabetes, dyslipidemia, and hypertension.

The detrimental effects of type 1 and 2 diabetes mellitus (T1- and T2DM) on cognitive functioning were also studied [11,12]. The authors of a previous paper reviewed evidence from neurocognitive testing and suggested that cognitive disorders should be listed among many other complications, due to diabetes, retinopathy, neuropathy, nephropathy, and cardiovascular disease [11]. Among other metabolic disorders, cardiorenal metabolic syndrome occupies an important role in bringing about diabetic cognopathy [12]. The authors indicted the potential causes of diabetic cognopathy in cardiorenal metabolic syndrome. In their conclusion, they affirmed that cardiorenal metabolic syndrome, obesity, and T2DM were associated with increased risks of vascular dementia, Alzheimer’s disease, mixed dementia, and diabetic cognopathy. In addition to these disorders, multiple metabolic toxicities promote oxidative stress and inflammation. These effects result in endothelial dysfunction, which translates into diabetic cardiomyopathy and diabetic nephropathy. Finally, these modifications may also contribute to subsequent brain remodeling as time progresses. Moreover, insulin resistance and reactive oxygen species are closely involved in the development of end-organ remodeling and disease [12].

Central obesity and insulin resistance are associated with hyperleptinemia, coupled with leptin resistance and reduced leptin transport across the blood–brain barrier (BBB) and into the hypothalamus and hippocampus [13,14,15]. The fasting leptin between obese and lean subjects demonstrated an inverse correlation, with cerebral grey matter volume. Moreover, it is important to highlight that altered leptin transport across the BBB, due to hypertriglyceridemia, paired with peripheral leptin resistance and central leptin resistance, could play a crucial role in the development and progression of obesity [15].

People with obesity can conceivably build-up a condition known as leptin resistance, which implies that their cerebrums no longer react to leptin. The absence of reaction brings about cravings to keep eating, expanding muscle to fat ratio levels, along with leptin levels [16]. This hormone attempts to hold glucose levels within proper limits, and may consistently rise as one puts on weight, inevitably prompting opposition, and the improvement of T2DM. Consequently, high levels of low-density lipoproteins (LDL), blood pressure, smoking, and T1DM define the risk factors of cardiovascular diseases (CVD). Moreover, insulin resistance and leptin can lead to CVD events, but further research needs to be conducted.

In this work, we presented the major effects of obesity-induced inflammation on CVD patients. This review focused on the association among cardiovascular disease, insulin resistance, and molecular mechanisms of obese individuals.

## 2. Methods

### 2.1. Search Strategy

The paper search was conducted in December 2020 through the databases PubMed (Medline), Scopus (EMBASE), and Cumulative Index to Nursing and Allied Health Literature (CINAHL). Keywords, such as “obesity”, “effects of obesity”, and “insulin resistance”, were used during the search. Each word was paired with the terms “CVD”, “cardiovascular”, “CVD population”, and “blood pressure”. Considering three independent literature databases, the search terms used for CINAHL and Scopus slightly varied from the search terms of PubMed. Boolean operators along with quotation marks were used to understand the relationship between obesity and CVD patients.

### 2.2. Inclusion and Exclusion Criteria

The inclusion criteria to extract studies from the libraries: language, availability of full article version, original study, and research interest. In the first stage, all authors carefully read the title and abstract of each paper for duplicates. Articles that did not match the inclusion criteria and study objectives were excluded. Later, the articles were equally distributed among the authors for a quality check.

### 2.3. Quality Evaluation

To conduct a quality evaluation of filtered papers in the first phase, the Newcastle–Ottawa Scale (NOS) was employed [17]. NOS scores are highly used to understand the nature of non-random studies based on factors of structure, convenience, and substance, and assess the individual study quality that is suitable for review studies. Quality assessment was evaluated based on final NOS scores of each study, such as poor (if score 0–3), modest (4–6), and excellent (7–9). Studies that passed the quality test of NOS ≥ 7 were considered for this review.

## 3. Results

### 3.1. Search Outcomes

**Figure 1:**
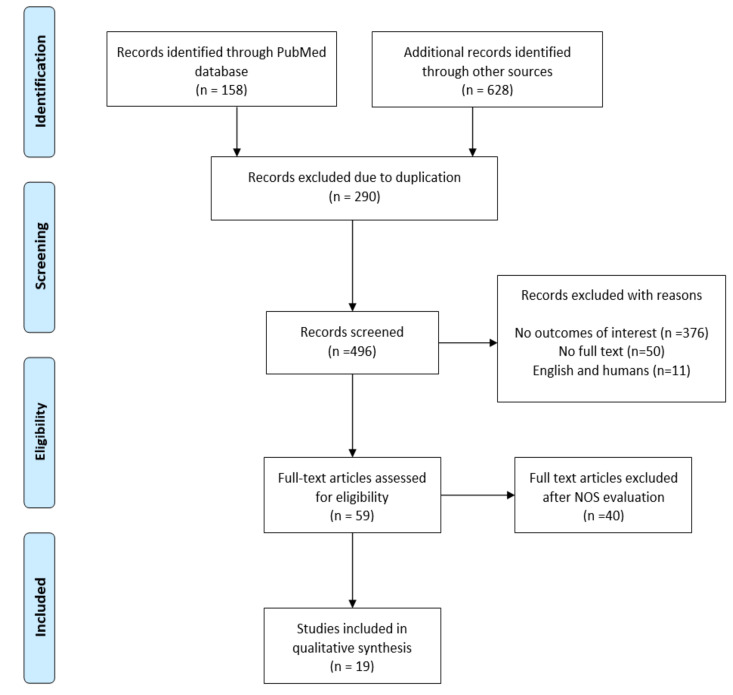
The study selection flow chart.

The search displayed 786 studies, and article evaluation was conducted in different steps based on preferred reporting items for systematic reviews and meta-analyses (PRISMA) guidelines [18]. A total of 290 articles were excluded because of duplication. At the first stage of filtering, 59 items satisfied the inclusion criteria and underwent further qualitative assessment. Ultimately, 19 studies were chosen for insight reviews, and a complete description of the study selection can be observed in Figure 1.

### 3.2. Study Characteristics

Table 1 presents an overview of the reviewed studies on obesity and inflammation, obesity-caused CVD, and its low-grade inflammation effects.

#### 3.2.1. Obesity and Inflammation

El-Wakkad A. et al. evaluated the association between anti-inflammatory cytokines (IL-4 and IL-5) with central obesity [19]. The authors considered a total of 86 obese girls and categorized them into two groups per central obesity: accordingly, group I (43 study subjects) with waist-to-hip ratio less than 0.8, as a control, and group II (43 study subjects) with waist-to-hip ratio greater than 0.8, as one case (central obesity). As a result, in group two, there were significant increases of proinflammatory cytokines, TNF-alpha (*p*< 0.0001) and IL-1β (*p* < 0.0001). Moreover, anti-inflammatory cytokines IL-4 (*p* < 0.0001) and IL-5 (*p* < 0.0005) significantly increased in group II when compared to group I. The authors reported that there was a significant decrease in anti-inflammatory adiponectin and an increase in proinflammatory leptin levels in group II. The result showed that central obesity lowers adiponectin plasma levels via increasing proinflammatory adipokines, such as TNF-alpha, leptin, and IL-1β [19]. Obese people have a higher level of serum tumor necrosis factor alpha (TNF-α), interleukin-1 beta (IL-1β), interleukin -6 (IL-6); these all are produced by macrophages derived from adipose tissue.

Adipocytes are the main sources of leptin among proinflammatory and adiponectin of anti-inflammatory adipokines, where levels increase and decrease, respectively, in obese individuals with MetS. The study by M.D. Borges and colleagues reported that leptin was positively correlated with BMI (BMI), abdominal circumference, insulin, IL-6, and TNF-α in obese study subjects. Moreover, the same study reported that leptin was associated negatively with HDL-C and adiponectin in obese individuals [20]. This indicates that obesity can change the lipid profiles of individuals by increasing and reducing the levels of pro-inflammatory and anti-inflammatory adipokines, respectively. Larsson A. et al. (2015) reported that IL-6 and TNF-alpha play a critical role in obesity-induced inflammation and their levels increase parallel to the rise of BMI [21]. IL-2 is one of the proinflammatory cytokines produced by activated CD4+ and CD8+ T-cells that play an important role in regulatory T-cells. However, obese people have lower IL-2 levels [22].

TNF-alpha is one of the major pro-inflammatory factors, secreted by endothelial cells and infiltrating immune cells. It modulates the adaptive and innate immune response during inflammation. In contrast, a study revealed that individuals with increased BMI and white adipose tissue (WAT) have increased TNF-α and IL-6 levels [23]. Another study reported that IL-6 and IL-8 secretions increased in obese individuals through cross-talk of chondrocytes and synovial fibroblasts related to increased leptin [24]. Beta cells appear to play a key role in inflammation in obesity because they can secrete IgG and can, consequently, directly activate macrophages [38]. The study in patients with modest obesity and early metabolic dysfunction reported that TNF-alpha in visceral adipose tissue was higher in the obese (BMI > 25 kg/m^2^) [25]. The same study reported that circulating levels and tissue expression levels of adipokines and proinflammatory markers changed significantly in obese subjects. Hence, the authors’ findings indicate that inflammatory biomolecule production changes precede increased inflammation or deactivate the macrophage phenotype of adipose tissue in obesity-related MetS, such as insulin resistance [25]. Engeli S. et al. (2003) revealed that plasma levels of IL-6 and high sensitive C-reactive protein (hs-CRP) and adipose tissue gene expression of IL-6 and TNF-α significantly increased with BMI; the study also reported that the increased plasma levels of IL-6 and hs-CRP were strongly correlated with decreased plasma levels of adiponectin [26]. Thus, the inverse association between plasma adiponectin and hs-CRP levels may indicate that reduced adiponectin production contributes to systemic and vascular inflammation in obesity [26].

A prospective study reported an association between obesity and various inflammatory markers [27]. Moreover, another population-based study (*n* = 740) revealed that abdominal obesity correlated with IL-6 and CPR [28]. Benbaibeche H. et al. (2020) reported a negative association of miR-146a and miR-21 concentrations with IL-6, TNF-a, and CD36 (cluster determinant 36) in obese subjects. The same study also reported that the downregulation of miR-146a and miR-21 was linked with the upregulation of the inflammatory state in obese [29].

Among the different studies that demonstrated the association between obesity and inflammation, there was a report that analyzed the obesity paradox in ischemic stroke [36]. In this paper, the authors designed a prospective case-control study in patients with acute ischemic stroke categorized into obese (BMI ≥ 30 kg/m^2^) and non-obese (BMI < 30 kg/m^2^). The results were quite surprising, as one of the primary endpoints, namely the functional outcome at three months, evidenced no difference in this endpoint (*p* = 0.882), although greater recovery of neurological impairment was seen in the obese group than the non-obese group. Several factors were associated with poor prognosis, obese patients did not evolve worse than non-obese after ischemic stroke, but experienced greater recovery in regards to neurological impairment. The role of obesity in cerebrovascular and cardiovascular disorders should be studied further, particularly from an inflammatory point of view.

#### 3.2.2. Obesity in Cardiovascular Diseases (CVD): Low-Grade Inflammation Effects

Obesity is a pro-inflammatory state associated with an increased risk of cardiovascular disorders [30]. Inflammation induced by obesity predisposes to a low-grade inflammatory state, resulting in various metabolic deregulations, such as increased insulin resistance and endothelial dysfunction, precipitating CVD. Mahabadi A.A. et al. (2009) reported that visceral adipose tissue and pericardial fat were associated with coronary heart disease (CHD) and myocardial infarction [31]. This is because adipokines are produced by adipose tissue, which is also epicardial and a visceral location, contributing to adverse cardiometabolic complications.

A study found that obese patients in the highest BMI quartile (BMI: 40.3–61.2 kg/m^2^) had significantly higher CRP levels (4.83 μg/mL vs. 3.03 μg/mL; *p* = 0.033) and higher leptin levels (44.97 ng/mL vs. 24.64 ng/mL; *p* = 0.042) than patients in the lower quartile of BMI (BMI, 28.6–32.4) with heart failure, type 2 diabetes mellitus, and MetS [32]. Thus, obesity-related inflammation occurs mainly in adipose tissue due to alterations in metabolic homeostasis, which leads to increased secretion of pro-inflammatory cytokines and activation of inflammatory signaling pathways in the body. Moreover, the increased CPR levels in obese subjects with MetS and the reduced correlation with weight loss are symptomatic of a link between CPR and obesity-related risk for cardiovascular diseases [33,34]. Therefore, CPR level increases with parallel BMI, a low-grade marker of inflammation. J. Lopez-Sandoval S. et al. (2018) reported that a higher level of inflammatory markers and low plasma adiponectin levels in obese adolescents can be at high risk for the development of CHD and T2DM [35]. A study reported that obesity was a low-grade chronic inflammatory state characterized by increased levels of pro-inflammatory cytokines and acute-phase proteins in the circulation [20]. Thus, the increased secretion of proinflammatory cytokines in obese individuals contributes to inflammation-related MetS, such as insulin resistance and other CVD risk factors in obesity.

The correlation between cardiovascular disorders and cognitive decline was evaluated through N-terminal pro-B-type natriuretic peptide (NT-proBNP) determination [37]. The results evidenced that even a subclinical CVD may be associated with dementia, particularly vascular dementia. Therefore, the measurement of this peptide could be considered a useful marker of imminent cognitive decline and dementia in absence of clinical CVD. A possible direct relationship between obesity and cardiovascular disease was evaluated by many authors from a different point of view. In general, obesity was considered a necessary condition, but not sufficient to develop cardiovascular disorders [38,39]. Other conditions that should be present include blood pressure, glucose (diabetes), dyslipidemia, insulin resistance, and inflammation. On the other hand, when we analyze obesity, we should take into account not only BMI, but also waist measurement and waist-to-hip ratio. In a prospective epidemiological analysis, the results demonstrated that recurrent CVD was common in the subjects with low BMI, increased waist measurement with BMI, high visceral/ectopic fat, low cardiovascular fitness activity, and low lean body mass.

These data suggest that obesity both directly and indirectly may negatively influence cognitive functions. Moreover, the inflammatory processes, due to obesity and cardiovascular diseases, including coronary heart disorders, lead to further complications in the peripheral organs and in the central nervous system. However, to follow all-embracing reasoning, we should keep in mind that the anatomical modifications of arterial vessels during aging or pathological processes play a crucial role in heart failure and stroke, with or without obesity involvement. The role of arterial stiffness in cognitive impairment and dementia was reviewed. Lutia M.F. et al. evidenced that, unfortunately, in contrast to blood pressure monitoring, large artery stiffness is not typically measured in routine clinical practice [40]. Therefore, relevant data on prevalence and incidence of stiffness, according to age groups, gender, or ethnicity remains scarce, as so is data on the use of arterial stiffness measures for risk assessment. It would be extremely useful to organize some trials to study the effects of the products, which decrease arterial stiffness, on cognition decline as primary or secondary endpoints.

## 4. Discussion

This study demonstrated the association between obesity and cardiovascular disease mediated by inflammation, which can also be observed in animal and epidemiological studies. An increase in visceral adipose tissue deposition induces chronic local and systemic inflammation, which mediates most obesity-related complications. The inflammation of the adipose tissue is characterized by the infiltration of activated M1 macrophages, leading to the production of reactive oxygen species (ROS) and release of pro-inflammatory cytokines, such as interleukin 6 (IL-6) and tumor necrosis factor-alpha TNF-α [41]. Chronic inflammation has been linked to the development and progression of cardiovascular diseases, cognitive decline, and cancer, suggesting an important pathophysiological role [42].

Cardiovascular diseases, such as heart failure and coronary heart diseases, are often among obese people. In the USA, every year 400,000 novel cases are diagnosed because of cardiovascular breakdown, and around 3 million people present side effects of heart failure, making it the new cardiovascular pandemic of the 21st century. It was suggested that the occurrence of obesity could be “halfway” because of the expanded rate of heart failure in recent times [43]. Moreover, these medical emergencies are dependent on the mechanical and epidemiological proofs that are associated with them.

The recurrence of cardiovascular breakdown is growing and it is one of the significant reasons for global deaths in developed countries, with about 3% prevalence [44]. A standard correlation can be seen between obesity and reasons for CVD. When compared to normal BMI individuals, overweight and obese candidates can experience earlier phases of heart disease (by approximately 10 years). The results of study [45] mentioned that the risk of cardiac arrest can be increased by 5% for men and 7% for women, with a BMI increase of 1 kg/m^2^. Similarly, 32–49% of obese people and 31–40% of overweight individuals have a high number of heart failures. The span of morbid obesity is firmly connected to the advancement of a cardiovascular breakdown following 20 years of obesity; 70% are at the risk for CVD after 20 years and 90% are at risk for CVD after 30 years [46]. The importance of obesity is shown by the Framingham Heart Study that highlighted the pathogenic role of obesity and heart failure onset in 11% of men and 14% of women [45]. The underlying changes of the heart seen in obesity alone add to a physiological crumbling in myocardial capacity, which is often called obesity cardiomyopathy.

**Figure 2:**
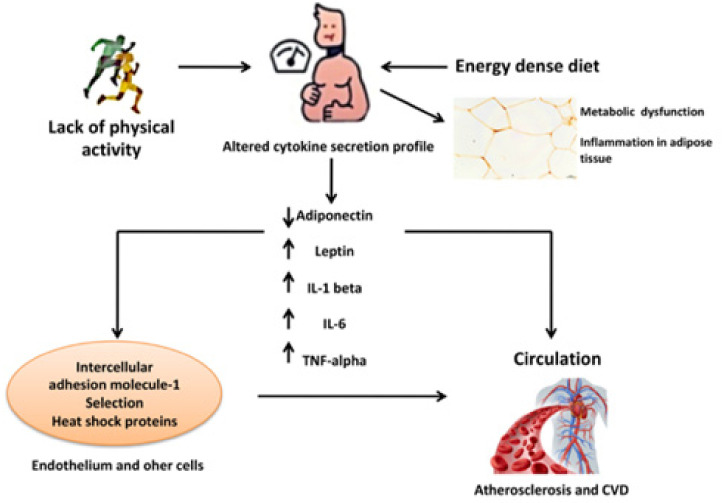
Cardiovascular disease (CVD) is related to inflammatory processes in obesity; modified from Mathieu et al., 2010 [49].

However, it is proven that obese people have a double risk for heart failure when compared with people of a normal BMI [47]. Patients with progressive obesity can suffer from heart failure without having a proper diagnosis for left ventricular brokenness, and are analyzed as having weight-related cardiomyopathy. Because of inflammatory symptoms, adipocytes appear to stimulate the production and release of inflammatory factors, such as TNF-α, plasminogen activator inhibitor-1 (PAI-1), IL-6, IL-8, IL-10, IL-15, and IL-1β, leukemia inhibitory factor, SAA3, haptoglobin, hepatocyte development factor, supplement factors B, D, C3, macrophage movement inhibitory factor, prostaglandin E2, and potential provocative modulators, such as adiponectin, leptin, and resistin [48]. However, many of them are confined to paracrine and autocrine impacts, and a portion of these cytokines produced from adipocytes and macrophages of residents in adipose tissue can contribute to systemic inflammation (Figure 2).

A series of research reports highlighted the potential mechanisms by which the development of fat deposition can cause inflammation [50,51,52]. However, the real picture is far behind in making valid conclusions, except in some high-risk factors. Because obesity is too unpredictable, even considered a single factor, the immediate rise in such a little time frame implies an important role of lifestyle and epigenetic factors, including food consumption and active work in the pathogenesis of obese individuals. Uncorrected obesity, alone, is recognized as a risk factor for diabetes mellitus, hypertension, dyslipidemia, and cardiovascular diseases. An inactive way of life alongside excessive caloric intake has been considered one of the fundamental drivers of overweight and metabolic syndrome [53]. Obesity possesses a significant negative effect on the heart, as a result of autonomic incitement and excess fat gathering in and around the heart [54]. Obesity prompts cardiac remodeling (hypertrophy), ventricular contractile brokenness, and interstitial fibrosis [54].

An obese or overweight population may show strikingly different profiles of CVD risk factors based on their body fat distributions. Excess abdominal fat tissue, independent of BMI, has been related to atherogenic and diabetogenic factors, for example, increased triglycerides, insulin resistance, and apolipoprotein B levels, an increased portion of low- and very-low-density lipoprotein [55]. On the other hand, low levels of fat tissue and general obesity are related to normal risk of metabolic profile. Moreover, it is mandatory to highlight an epicardial adipose tissue (EAT), which has a significant fat portion related to obesity and CVD [56]. Since this tissue is straightforwardly lying on the outside of the myocardium and in direct contact with coronary vessels, this portion can be referred to as extraordinary perivascular adipose tissue. The correlation between the density of EAT, the occurrence of CVD, and metabolic syndrome is broadly acknowledged in [55]. Few studies discussed the cross-talk between EAT and cardiomyocytes [57]. EAT secretory products initiate insulin resistance; alteration in contractility and calcium flood in cardiomyocytes by the responsible adipokines still need to be addressed [58].

## 5. Therapeutical Strategies

### 5.1. Pharmacological Intervention

The development of pharmacological agents to reduce visceral obesity is difficult due to various potential side effects [59]. Regulatory authorities approved dexfenfluramine, sibutramine, and rimonabant for the treatment of visceral obesity and overweight. However, they were removed from clinical use due to their various side effects. Other experimental drugs that may probably reduce visceral fat may be risk factors for CVD. Peroxisome proliferator-activated receptor gamma has critical roles in various cellular functions, in glucose, and lipid metabolism [60]. PPAR-γ agonists have cardiovascular protective action due to the effects on the reduction of blood pressure and improvement of endothelial function [42]. It was suggested that growth hormone therapy decreases visceral adiposity and improves lipid profile in adults with obesity, with effects at the level of the cardiovascular system.

Metformin, a biguanide anti-hyperglycemic agent, represents the first drug in the treatment of overweight diabetes patients [61]. Metformin reduces liver glucose production, increases cells insulin sensitivity, increases glucose uptake, and reduces serum insulin level. The drug shows cardiovascular protective effects independent of the reduction of glycemia. It increases eNOS production, attenuates ER stress-induced mitochondrial dysfunction, and reduces cardiac injury through ER stress [42].

Non-steroidal anti-inflammatory drugs (NSAIDs) reduce inflammation via cyclooxygenase (COX) inhibition and reduction of prostaglandin levels [42]. Elevated levels of COX represent a sign of chronic inflammation. In obese subjects, the inflammatory reduction induced by NSAIDs determines a reduction of platelet aggregation and vasoconstriction effects on visceral obesity with an adipose tissue reduction [62]. Different statins are effective in lowering cholesterol and could represent important compounds in the prevention of primary and secondary cardiovascular disease. Statins improve endothelial function and induce plaque stabilization, but also decrease chronic inflammation and oxidative stress, which represents another possible mechanism in the prevention of cardiovascular disease [63]. Moreover, a preclinical study demonstrated that a moderate- and low-dose of Atorvastatin induced a significant attenuation of cognitive impairment, due to a high-fat diet, through its antioxidant and anti-inflammatory activity, related to the activation of silent information regulator-1 (SIRT1) [64].

In high-fat diets (HFDs), obese mice, simvastatin, could reduce HFD-induced structural abnormality, neuronal injury, serotonergic system disturbance, and pro-inflammatory (IL-1β, IL-6, TNF-α) cytokine overexpression in the hippocampus. These results indicate that simvastatin may be a promising treatment for HFD-induced depression-like behavior during the adolescent period through neuroinflammatory mechanisms [65]. Ezetimibe is another lipid-lowering drug that limits the absorption of cholesterol from the gastrointestinal tract epithelial. The study suggests that ezetimibe has an effect on adiponectin and decreases insulin resistance in metabolic syndrome patients, inducing adipose tissue reduction [66]. The drug can reduce the blood level of TNF-α in patients with hyperlipidemia, reducing the risk of cardiovascular disease [42]. In the hearts of obese rats, ezetimibe decreased the expression of inflammatory molecules (IL-6, intercellular adhesion molecule-1, and vascular cell adhesion molecule-1), indicating that ezetimibe may improve myocardial remodeling by inhibiting inflammation [67]. Among novel strategies to treat obesity-associated inflammation, there is growing interest in the use of foods with nutraceutical properties, useful to treat and prevent human diseases [68].

### 5.2. Non-Pharmacological Interventions

Berries have been extensively studied for their anti-inflammatory properties, which may translate to beneficial health outcomes. Preclinical rodent and cell culture studies provide robust evidence that berries (blackberries, raspberries, strawberries, blueberries) and their bioactive components have beneficial effects—and not only on inflammation. Berries contain an abundance of bioactive compounds of the flavonoid family (anthocyanidins, flavanols, and flavones), phenolic acids, tannins, and stilbenes were shown to inhibit inflammation and reduce reactive oxygen species. Therefore, berries represent an intriguing possibility for the treatment of obesity-induced inflammation and associated comorbidities. A recent review highlighted that the primary mechanisms mediating the anti-inflammatory effects of berries include a reduction in nuclear factor kappa light chain enhancer of activated B cells. This signaling may be secondary to reduced oxidative stress, a downregulation of toll-like receptor 4 (TLR4) signaling, and an increase in nuclear factor erythroid 2–related factor 2 (Nrf2) [69].

Similar to berries, cherries are a rich source of polyphenols and vitamin C, which have antioxidant and anti-inflammatory properties [70]. Human studies highlighted that consumption of cherries decreased markers for oxidative stress and inflammation with control of the level of serum CRP, IL-6, TNF-α, and monocyte chemoattractant protein-1 (MCP-1). Cherries can reduce cardiovascular risk factors, such as blood pressure and glycemia level [71]. On the contrary, limited numbers of published papers also indicate beneficial effects of consuming cherries on blood lipids, diabetes, and cognitive function [71]. A recent study demonstrated in obese subjects, as individuals at risk for chronic metabolic inflammation, a significant reduction in pro-inflammatory MCP-1, a trend for reduction of TNF-α, a decrease of 5% of erythrocyte sedimentation rate (ESR) after consumption of tart cherry juice for four weeks [72]. Recently, in preclinical studies in rats with diet-induced obesity (DIO), the supplementation of tart cherry juice and seeds reduced the neuroinflammatory process, liver steatosis, inflammation, and adipose gene transcription in visceral adipose tissue [73,74,75,76].

Another study suggested that berry fruit consumption has a significant role in the prevention and treatment of most risk factors associated with obesity and metabolic syndrome, and its cardiovascular complications in the human population [77]. In metabolic syndrome, evidence suggests that consumption of berries, whose distinctive nutritional characteristics are phenolic compounds, has the potential to affect several metabolic abnormalities related to the development of CVD, such as hyperglycemia, high levels of triglycerides, low HDL-cholesterol levels, hypertension, and endothelial dysfunction [77].

Reducing BMI by 1–3 kg/m^2^ was associated with a 2–13% lower risk of CVD events and mortality [78]. In a prospective study that compared the in-takes of 16 common fruits, the highest blueberry intake was associated with the least weight gain [79]. In this study, in a cohort of 124,000 individuals, among six classes of flavonoids, a higher anthocyanin intake had the strongest association with less weight gain (−0.1 kg per 10 mg anthocyanins) [79].

Anthocyanins and flavonoid compounds present in different fruits showed antioxidant and anti-inflammatory effects. If it is true that obesity causes cardiac and cerebrovascular disease through the development of hypertension, T2DM mellitus, dyslipidemia due to inflammation, and oxidative stress, the use of antioxidant compounds may help neutralize the free radicals and protect from organ damage. Ascorbic acid (vitamin C) may help in preventing oxidative damage, preventing non-enzymatic glycosylation of proteins, and enhancing arterial dilation, affecting nitric oxide release. Moreover, it decreases lipid peroxidation and alleviates inflammation [80]. The possible beneficial effects of ascorbic acid on obesity-related mechanisms include the modulation of adipocyte lipolysis, the inhibition of glucose metabolism with a decrease of hyperglycemia and glycosylation, and leptin secretion inhibition with a reduction of the inflammatory response in obese-diabetic models [81].

However, there was a recent review on the weakness of the current evidence based on the effects of vitamin C supplementation on markers of CVD risk. The authors concluded that there is limited evidence that some population subgroups, such as the elderly, subjects with lower vitamin C status at baseline, obese, and patients with high CVD risk, may be more responsive to vitamin C supplementation, and offer opportunities for tailored nutritional interventions to improve cardiometabolic health [82].

Another component of fruits with antioxidant properties is represented by resveratrol, highly concentrated in grape skin. Red wine is the most concentrated food source of resveratrol found in the human diet, in particular in the Mediterranean diet. Resveratrol has a protective effect on brain aging of the elderly. Its role on the microglial cells plays a central role in neuroinflammation [83]. Supplementation with resveratrol in rodents protected animals against HFD-induced body weight gain and obesity. It increased energy expenditure, which was partly mediated by stimulating intracellular mitochondrial functions (fatty acid oxidation) in adipose tissue and by fatty acid synthesis suppression [84]. In mice, resveratrol reduced HFD-induced inflammation of WAT by downregulating proinflammatory cytokines, TNF-α, IFN-α, IFN-β, and IL-6 [85]. In obese subjects, resveratrol exhibited a vascular protective effect, mimicking caloric restriction [86,87,88].

Recent experimental studies have found that resveratrol offers a variety of benefits that include both anticarcinogenic and anti-inflammatory effects, in addition to the ability to reverse obesity, attenuate hyperglycemia and hyperinsulinemia, protect the heart, endothelial function, and increase the life span [89]. Multiple molecular targets are associated with the cardioprotective capabilities of resveratrol based on its ability to modulate multiple cell signaling molecules, such as cytokines, caspases, matrix metalloproteinases, nuclear factor-κB, Notch, intercellular adhesion molecule, vascular cell adhesion molecule, peroxisome proliferator-activated receptor-γ coactivator 1α, insulin-like growth factor 1, insulin-like growth factor-binding protein 3, Ras association domain family 1α, pAkt, vascular endothelial growth factor, cyclooxygenase 2, and nuclear factor erythroid 2 like [90]. Moreover, resveratrol directly upregulates antioxidative capacity by increasing antioxidant genes, such as heme oxygenase-1, superoxide dismutase, catalase, and glutathione, and directly modulates the cardiac mitochondrial function in cardiac pathologies related (or not) to obesity [91].

Omega-3 fatty acids are dietary essential polyunsaturated fatty acids (PUFAs) [92]. The plant form of omega-3 fatty acids is a short-chain fatty acid, a-linolenic acid, obtained from plant oil including leafy vegetables, walnuts, soybean oil, canola oil, and flaxseed oil [93]. The marine forms of omega-3 fatty acids are long-chain fatty acids, such as docosahexaenoic acid (DHA), and eicosatetraenoic acid (EPA). They are obtained from seafood, fish, and algae [94].

An inverse relationship between omega-3 fatty acids, inflammation, obesity, and CVD has been demonstrated. Leptin, adiponectin, and resistin represent a set of hormones associated with the development of CVD, obesity, T2DM, and insulin resistance, and are modified in obese/overweight people compared to normal-weight individuals. Omega-3 PUFAs were shown to decrease the production of inflammatory mediators, having a positive effect on obesity and T2DM. Moreover, they significantly decrease the appearance of CVD risk factors [95].

Based on the review studies, the role of omega-3 on inflammation presents different pathways. Omega-3 FAs can respond to inflammation in CVD and atherosclerosis through direct and indirect mechanisms. A direct mechanism through which omega-3 fatty acids decrease inflammation includes their rapid effect on the regulation of transcription factors and indirect action, including the production of three- and five-series eicosanoids, inflammation-resolving lipid mediators, and suppression of acute-phase reactants [96]. Moreover, omega-3 fatty acids inhibit inflammation by reducing TNF-α, IL-6, C-reactive protein, and by increasing the three- and five-series eicosanoids, lipoxins, resolving, and protections that essentially derived from omega-3 fatty acid. A clinical study highlighted different mechanisms of action for omega-3, which may involve the decrease of expression of the inflammatory genes and increase the production of anti-inflammatory eicosanoids in visceral subcutaneous adipose tissue [96]. Moreover, serum interleukins and protein-C reactive decreased in patients supplemented with omega-3, improving the endothelial functions. The supplementation restored endothelial-dependent flow-mediated dilation in hyperlipidemic patients [96].

Most CVD risk factors, including aging, obesity, dietary patterns, and a sedentary lifestyle, were shown to induce gut dysbiosis. Dysbiosis is associated with intestinal inflammation and reduced integrity of the gut barrier, which may facilitate the development of CVD [97]. The World Health Organization defines probiotics as live microorganisms that, when consumed in adequate amounts, have a positive influence on the individual’s health. Another study presented the effects of probiotics, i.e., lower low-density lipoproteins (LDL)-cholesterol and improving the LDL/high-density lipoproteins (HDL) ratio. Moreover, lower blood pressure, inflammatory mediators, blood glucose levels, and body mass index probiotics have the scope to be developed as dietary supplements with potential cardiovascular health benefits [98].

Probiotics have specific activity against the factors causing CVD, such as oxidative stress, inflammation, hypercholesterolemia, and hypertension [99]. A recent meta-analysis showed a highly significant reduction in cardiovascular risk factors such as systolic and diastolic blood pressure associated with T2DM. Moreover, the effects on the reduction of total cholesterol LDL-C were associated with hyperglycemia, increasing the level of glycated hemoglobin, hypertension, hypercholesterolemia, yoghurt intake, and less than 1.5 months probiotic intake. Additionally, the probiotic supplement had a beneficial effect in reducing BMI associated with obesity, higher dosage intake of probiotics, and more than 1.5 months duration of intake [100].

**Figure 3:**
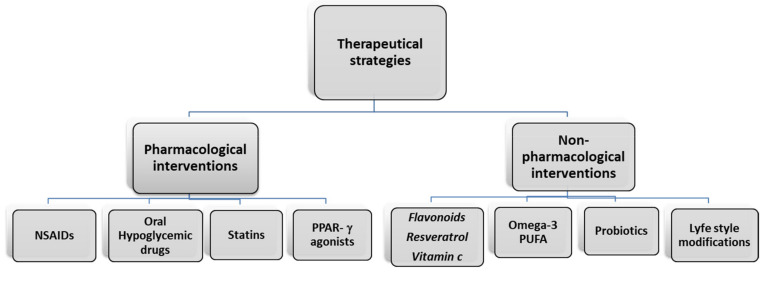
Pharmacological and non-pharmacological interventions to prevent CVD complications in obesity. Peroxisome proliferator-activated receptor gamma (PPAR-γ); non-steroidal anti-inflammatory drugs (NSAIDs).

There are limited data to draw deeper meaningful conclusions beyond simple associations, which carries the risk of misinterpretation or assigning excess value to expected results when translating animal protocols into human trials. Finally, it is important to consider the role of the factors linked to lifestyle, in particular correct diet and exercise, which can directly modify the risk of disease development. In particular, regular exercise is an important lifestyle factor that not only combats obesity and related metabolic diseases, but also reduces the risk of cerebro–cardiovascular disease [101,102]. Exercise also plays a pivotal role in the prevention of neuroinflammation [103]. It has anti-inflammatory effects in the periphery by reducing markers of inflammation induced by obesity [103]. Specifically, several studies have demonstrated that aerobic and-or resistance training is effective at decreasing circulating inflammatory markers, such as IL-6, IL-1β, and TNF-α, increasing circulating anti-inflammatory markers, such as IL-10 and IL-4 in humans [104]. At the same time, it is well established that exercise can improve insulin sensitivity and glycemic control in healthy individuals as well as obese and diabetic subjects [105]; it represents an important instrument to prevent the development of cardiovascular disease. In fact, current cardioprotective therapies must include exercise and lifestyle interventions in addition to pharmacologic agents [106]. Different possible therapeutical strategies are summarized in Figure 3.

## 6. Conclusions

This study presents a review of studies in regards to the impact of obesity inflammation on CVD. Overweight–obesity is associated with an increased risk of CVD, as well as other health issues, such as diabetes, hypertension, and insulin resistance. The assessment of biochemical parameters, such as leptin and inflammatory cytokines in CVD, show that leptin is largely correlated with BMI, and hyperleptinemia can be unfavorable for CVD prognosis. Moreover, the inflammation of adipose tissue and systemic inflammation could induce an endothelial alteration that represents the pathological basis of CVD. Therefore, the identification of drugs or other natural molecules able to contrast inflammation and leptin secretion may represent a useful therapeutical approach to treat obesity-related diseases.

## Figures and Tables

**Table 1:** Summary of the results and characteristics of selected studies.

No	Study Design	Sample Size	Main Outcomes	Ref.
1	Cross-sectional	86 patients	Positive correlation between waist-to-hip ratio and tumor necrosis factor-alpha (TNF-α) (r = 0.559, *p* < 0.001), interleukin-1beta (IL-1-β) (r = 0.435, *p* < 0.004), IL-4 ((r = 0.509, *p* < 0.001), IL-5 (r = 0.550, *p* < 0.005), leptin ((r = 0.331, *p* < 0.03), and negative correlation with adiponectin (r = −0.410, *p* < 0.006) in adolescents with central obesity.	[19]
2	Cross-sectional	30 patients	Inverse correlation between adiponectin with body mass index (BMI), abdominal circumference, cholesterol LDL-C, IL-6, TNF-α, and leptin, and a positive correlation with cholesterol HDL-C in obese individuals. Leptin positively correlated with BMI, abdominal circumference, insulin, IL-6, TNF-α, and LAR, and negatively correlated with cholesterol HDL-C and adiponectin in obese subjects.	[20]
3	Clinical trial	89 fluid samples	The negative association between BMI and inflammatory markers.	[21]
4	Case-control	40 patients	The obese chronic obstructive pulmonary disease (COPD) group had lower levels of IL-2 (*p* = 0.01) and higher interferon gamma (INF-γ) levels (*p* = 0.02) and IL-6 (*p* = 0.003) than lean COPD. Whereas lean COPD patients had higher CD25+ (*p* = 0.01), CCr5 (*p* = 0.04), and HLA-DR (*p* = 0.007) expression on T cell surface compared to overweight–obese COPD participants.	[22]
5	Follow up study	33 patients	Increased levels of C-reactive protein (CRP), TNF-α, triglycerides, homeostatic model assessment for insulin resistance (HOMA-IR), and fasting glucose, and a decreased level of high-density lipoprotein (HDL)-cholesterol were found in obese (BMI > 40 kg/m^2^) compared with the healthy individuals (BMI < 24.9 kg/m^2^).	[23]
6	Cross-sectional	56 patients	Peripheral blood or local lymphocytes did not differ between obese and normal-weight patients with hip osteoarthritis (OA). However, higher levels of IL-6 and IL-8 (*p* < 0.05) were detected in the synovial fluid of the obese OA patients.	[24]
7	Cross-sectional	51 female patients	Serum levels of adiponectin and leptin were significantly correlated with HOMA-IR and BMI. The levels of expression of monocyte chemoattractant protein-1 (MCP-1) and TNF-α in visceral adipose tissue were higher in the obese group (BMI ≥ 25). Moreover, the expression of mRNA MCP-1 in visceral adipose tissue was positively correlated with BMI (r = 0.428, *p* = 0.037).	[25]
8	Observational	65 postmenopausal women	Adiponectin plasma levels and adipose-tissue gene expression were significantly lower in obese subjects and negatively correlated with obesity-associated variables, including hs-CRP and IL-6.	[26]
9	Prospective study	85 patients	There was a negative association observed between obesity and adiponectin. Type 2 diabetes (T2D) patients have shown a significant correlation between plasma insulin, adipocytokines, and other inflammatory markers.	[27]
10	Cross-sectional	740 Type 2 diabetic patients	Abdominal obesity was significantly correlated with IL-6 (waist circumference (WC): r = 0.27, *p* < 0.001; sagittal abdominal diameter (SAD): r = 031, *p* < 0.001), CRP (WC: r = 0.29, *p* < 0.001; SAD: r = 0.29, *p* < 0.001), IMT (WC: r = 0.09, *p* = 0.013; SAD: r = 0.11, *p* = 0.003), and PWV (WC: r = 0.18, *p* < 0.001; SAD: r = 0.21, *p* < 0.001)).	[28]
11	Case-control	42 patients	microRNA-146a (miR-146a) and miR-21 concentrations were negatively correlated to IL-6, TNF-α, and CD36 in obese	[29]
12	Follow up study	200 patients	From baseline to Week 52 changes in serum leptin, adiponectin, IL-6, TNFα, CRP, PAI-1, vascular cell adhesion molecule-1(VCAM-1), and MCP-1 were measured in patients with T2D. At weeks 52, there was a 22% reduction in median serum IL-6 (95% CI: −34%, −10%) and a 7% increase in median serum TNFα (95% CI: 1%, 12%) with canagliflozin versus glimepiride.	[30]
13	Cross-sectional	1267 patients	Pericardial fat (odds ratio (OR) 1.32, 95% confidence interval (CI) 1.11–1.57; * p * = 0.002) and visceral adipose tissue (VAT) (OR 1.35, 95% CI 1.11–1.57; * p * = 0.003) were significantly associated with prevalent CVD in age–sex-adjusted models and after adjustment for BMI and waist circumference.	[31]
14	Cross-sectional	36 patients	A significant correlation was observed between CRP and leptin, CRP and BMI (BMI). Patients with the highest BMI quartile (BMI, 40.3–61.2) had higher CRP levels (4.83 μg/mL vs. 3.03 μg/mL; *p* = 0.033) and higher leptin levels (44.97 ng/mL vs. 24.64 ng/mL; *p* = 0.042) compared with patients in the lower BMI quartile (BMI, 28.6–32.4).	[32]
15	Randomized single-blind trial	120 premenopausal obese women	After 2 years of follow-up of obese women, BMI and serum concentrations of IL-6 (−1.1 pg/mL; *p* = 0.009), IL-18 (−57 pg/mL; *p* = 0.02) and CRP (−1.6 mg/L; *p* = 0.008) decreased, while adiponectin levels increased significantly (2.2 μ g/mL; *p* = 0.01) in the intervention group compared to controls (−4, 2; *p* < 0.001).	[33]
16	Follow up trails	83 Women	CRP was positively associated with BMI (r = 0.281, *p* = 0.01) and waist circumference (r = 0.278, *p* = 0.01). After 12 weeks, weight loss was 7.9+/−0.3 kg. CRP was significantly decreased by 26% (*p* < 0.001), and a correlation was observed between weight loss and the change in CRP (r = 0.309, *p* = 0.005).	[34]
17	Cross-sectional	83 patients	Obesity, dyslipidemia, IL-6, and CRP were significantly higher in the Insulin resistance (IR) group than in the non-IR group. Increased insulin levels, HOMA-IR, inflammatory markers, and triglycerides; while having lower HDL-C and adiponectin in obese adolescents than normal-weight adolescents.	[35]
18	Case control	98 patients	Differences in functional outcomes were not found for three months after stroke between obese and non-obese groups. Obese patients experienced a high reduction of body weight, and pro-inflammatory IL-6 levels were higher after strokes.	[36]
19	Follow up study	6040 participants	It was reported that the high value of N-terminal pro-B-type natriuretic peptide (NT-proBNP) is a major risk of dementia, excluding CVD patients and adapting risk factors. Higher NT-proBNP was cross-sectionally connected with more unfortunate executions in different psychological tests.	[37]

## Data Availability

Not applicable.
